# The Prevalence of Multidrug-Resistant Uropathogenic Bacterial Profile With Antibiotic Susceptibility Patterns Among the Community and Hospitalized Patients During COVID Waves

**DOI:** 10.7759/cureus.60613

**Published:** 2024-05-19

**Authors:** Newar D Shawkat, Najim Abdulla Yassin

**Affiliations:** 1 Department of Nursing, Akre Technical Institute, Akre University For Applied Sciences, Akre, IRQ; 2 Department of Medical Laboratory Technology, Technical College of Health-Shekhan, Duhok Polytechnic University, Duhok, IRQ; 3 Department of Microbiology, College of Medicine, University of Duhok, Duhok, IRQ

**Keywords:** covid-19, community and hospitalized patients, antimicrobial resistance, uropathogenic bacteria, urinary tract infections

## Abstract

Background and objective

Urinary tract infections (UTIs) are a common infectious disease affecting people of various ages and genders and are prevalent in different geographical locations. However, the way Gram-positive and Gram-negative (UTI) germs react to antibiotic treatment varies significantly. The coronavirus disease 2019 (COVID-19) pandemic has increased the frequency of secondary bacterial superinfection, leading to a spike in ongoing recommendations for antibiotic treatment, both therapeutic and preventative. In this study, we aimed to assess uropathogenic bacterial resistance and shed light on how COVID-19 epidemic waves influence the evolution of bacterial resistance.

Materials and methods

A cross-sectional study was conducted, assessing the different isolates of the uropathogen in all COVID-19 waves by using convenience sampling from August 2020 till the end of 2023. The VITEK-2 compact system employing industry-standard bacteriological tests to identify the bacteria and confirm their antibiotic susceptibility was utilized.

Results

Of the total 3877 patients, 381 (9.8%) and 3483 (89.8%) had positive and negative microbial growth, respectively. Of the 381 (9.8%) positive cases, 130 (34%) were male and 251 (65%) were female; 138 (43.3%) patients in the age range of 15-40 years developed sporadic UTIs attributed to Gram-negative bacteria. Alternatively, patients over 40 years had the highest prevalence rate (n = 180, 56.6%). The most common strains of Gram-negative and Gram-positive bacteria were *Escherichia coli (E. coli) *and *Staphylococcus aureus (S. aureus), *with* *278 (88.8%) and 13 (20.9%) cases respectively. People with Gram-negative bacteria who were not hospitalized were very resistant to trimethoprim/sulfamethoxazole (n = 219, 69.1%), cefotaxime (n = 193, 60.9%), ampicillin (n = 192, 60.6%), and amoxicillin/clavulanic acid (176, 55.5%). While high sensitivity to meropenem (n = 14, 4.4%) and imipenem (n = 13, 4.1%) was observed, hospitalized individuals had higher levels of resistance and great sensitivity to the same antibiotics. S. *aureus and Enterococcus faecalis (E*. *faecalis)* were commonly present. Hospitalized patients were less sensitive to benzylpenicillin, ampicillin, and oxacillin, and there was a big rise in resistance to cefoxitin in the community.

Conclusions

In this study, Gram-negative germs among females were predominantly observed with extremely high multi-drug resistance (MDR). The most effective antibiotics against Gram-positive germs included linezolid, vancomycin, and nitrofurantin, while those against Gram-negative bacteria were meropenem and amikacin. Clinicians should be regularly updated and informed about antibiotic selection through routine monitoring of uropathogenic bacteria's susceptibility. Moreover, we recommend changes to the local antibiotic policy regarding the selection of UTIs; further multicentric and high-volume studies are required to gain deeper insights into the topic.

## Introduction

According to the Centers for Disease Control and Prevention (CDC), urinary tract infections (UTIs) are the most predominant bacterial infections that require medical attention and they occur frequently in both community and nosocomial settings. The National Ambulatory Medical Care Survey has reported that UTIs alone account for approximately seven million patient visits to outpatient departments (OPDs) and as many as one million visits to emergency departments in hospitals, with an estimated 100,000 hospitalizations [1]. UTI ranks as the most common infection in both settings, especially with the rise in antibiotic resistance [2]. UTIs are classified as lower UTIs (cystitis, prostatitis) and upper UTIs (pyelonephritis) according to the anatomical function, the site of infection, and the complications from underlying illnesses. UTIs might manifest nonspecific, feverish signs and symptoms that impede treatment and raise the risk of severe consequences such as progressive renal failure, high blood pressure, and recurrent nephropathy. Moreover, they are significant contributors to morbidity in both outpatients and inpatients [3].

Patients who have received a confirmatory diagnosis of coronavirus disease 2019 (COVID-19) often require ICU admission, given the increased risk of grave consequences such as sepsis, shock, and severe renal injury [4]. Several studies have described a link between viral illnesses, including the flu virus and bacterial pneumonia, which occur as secondary illnesses (bacterial coinfections) in patients in ICUs [4]. Hospitalized patients, hospital equipment, and healthcare workers (HCWs) are highly susceptible to bacterial infections [5]. *Acinetobacter* spp., *Enterobacter* spp., *Enterococcus* spp., *Escherichia* spp., *Klebsiella *spp., *Pseudomonas* spp., and *Staphylococcus* species are often associated with hospital infections [6]. Frequent admissions to the ICU among critically ill patients with COVID-19 may be linked to a greater likelihood of bacterial coinfections [4].

Bacterial strains that spread in hospital settings and are often resistant to many drugs have been linked to 70,000 fatalities globally in 2019 [7], making patient treatment very challenging. Coinfection refers to the emergence of subsequent infection in COVID-19 patients during the period of identification or hospitalization [5]. Individuals are typically more susceptible to bacterial infections when excessive amounts of preventative or curative antibiotics are used [8]. To make informed decisions about prescription antibiotics for hospitalized patients, physicians must have access to a regularly updated hospital antibiogram [5]. Based on a current hospital antibiogram, local recommendations, and updated information about bacterial strains, doctors can select the appropriate antibiotic therapy to effectively eradicate dangerous bacteria [4].

The overuse of antibiotics in the treatment of UTIs is a major cause of antimicrobial resistance (AMR) [9]. An estimated 700,000 deaths are attributed to AMR globally; if the present trends persist, this number is projected to increase to about 10 million deaths a year by 2050 [7]. Since the start of the COVID-19 pandemic, there has been a rise in the use of antibiotics, accelerating the creation of germs resistant to these drugs [7]. These chronic infections spread across hospital settings, putting all patients at risk, regardless of their condition [7]. For physicians to prescribe antibiotics to hospitalized patients intelligently, they must have access to a regularly updated hospital antibiogram [4]. Along with revised hospital antibiogram utilization and local recommendations, clinicians can select the appropriate antibiotic medication for freshly discovered bacterial strains to successfully remove harmful microorganisms [10]. In this context, the current study aimed to investigate the prevalence of common local Gram-positive and negative uropathogen isolates with their antibiogram patterns among UTI patients admitted for fewer than 48 hours and those who presented to hospital OPDs during the COVID-19 pandemic in Akre District, Duhok Province, Iraq.

## Materials and methods

Study design and data collected

A cross-sectional study was conducted at Gulan General Akre Hospital, the principal regional center for treating COVID-19 patients in the area. Data on uropathogenic patients, including demographics (gender, occupation, age), admission status (ICU or non-ICU), and microbiology results (bacterial coinfection, susceptibility profiles), were collected anonymously over three years, from the peak of the first wave of COVID-19 in August 2020 to the end waves in 2023.

Ethical approval

All participants in this study either provided consent or waived it. The General Directorate of Health in Duhok Governorate issued approval for the study (no: 06052021-3-4, dated May 6, 2021). Informed consent was obtained from the respondents after explaining the objective of the study.

Sample collection, processing, and bacterial identification

All patients suspected of having UTIs were interviewed using a structured questionnaire including queries on demographics, patient type (hospitalized or outpatient), prior history of UTIs, and underlying medical problems. Only those with at least one clinical characteristic of UTI (such as dysuria, frequency, urgency, and pelvic pain) or a prior UTI history were deemed clinically suspected UTI patients and chosen for further testing. A total of 3877 midstream "clean-catch" urine samples were obtained from the same number of clinically suspected UTI patients. The samples were then immediately transported to the laboratory within not more than one hour and inoculated on blood agar and MacConkey agar by using standardized calibrated loops.

Purified colonies of isolates from cases with significant bacteriuria (≥105 colonies/ml) were identified using standard laboratory techniques, including morphological characteristics, Gram staining, rapid tests (such as catalase, oxidase, coagulase, and bile solubility), and biochemical tests (such as IMViC, TSI, urease, and nitrate reduction) [11]. A single isolate was chosen from each confirmed sample. UTI was diagnosed based on pyuria (≥5 leukocytes per high-power field on urine microscopic examination), clinical symptoms, and substantial bacteriuria. Separate bacterial colonies were inoculated on blood agar and MacConkey agar using standardized calibrated loops; after growth, these were subjected to Gram staining and identified by the VITEK-2 compact system (BioMérieux, France) using the appropriate VITEK-2 identification cards (GP/GN).

Antimicrobial susceptibility test

Antibiotic susceptibility testing was performed using a VITEK-2 Compact AST-GN and AST-GP (BioMérieux, France). Minimal Inhibitory concentration (MIC) breakpoints were assigned according to the Clinical and Laboratory Standards Institute (CLSI) guidelines [12]. The panel of Gram-negative antibiotics included cefuroxime, cefuroxime axetil, cefoxitin, ceftazidime, ceftriaxone, cefepime, aztreonam, entrapenem, imipenem, meropene, amikacin, gentamicin, ciprofloxacin, nitrofurantion, tigecycline, and trimethoprim/sulfamethoxazole. The identified Gram-positive bacteria were tested for gentamicin, ciprofloxacin, nitrofurantoin, tigecycline, and trimethoprim/sulfamethoxazole. The identified Gram-positive bacteria were gentamicin high-level resistance, and cefoxitin screen (present in AST Gram-positive card of VITEK), benzylpenicillin, levofloxacin, oxacillin, ampicillin, erythromycin, clindamycin, linezolid, daptomycin, teicoplanin, vancomycin, tetracyclin, tigecycline, nitrofurantoin, fusidic acid, mupirocin and trimethoprim/sulfamethoxazole. The results of susceptibility testing were reported as either "susceptible" or "resistant," according to CLSI guidelines [12].

Susceptibility data were presented as the percentage of resistant isolates to the total number of isolates recovered from each site or specimen for individual bacterial species. MDR organisms were defined as isolates that were non-susceptible to one or more agents in three or more antimicrobial categories, per CLSI guidelines [12].

Statistical analyses

Statistical analytical tools, including Excel and GraphPad Prism version 8.0.1, were used to analyze the profiles of antibiotic resistance, bacterial identification, and patient demographics. *P-*values less than 0.05 were deemed statistically significant. Statistical analyses were carried out using a t-test on GraphPad Prism. Whenever appropriate, the χ2 test or Fisher's exact test was performed to evaluate the antibiotic resistance patterns of bacteria during the three-year study period and the trend of antibiotic resistance. The proportion of resistant bacteria in positive UTI cases was also tested.

## Results

A total of 3877 urine culture results from outpatient and inpatient units were recorded. The overall prevalence of microbiological proof of infection was found in 381 (9.8%) samples. Among the 381 isolates obtained, the frequency of Gram-negative bacteria was higher than that of Gram-positive bacteria. We observed a linear increase in the incidence of each uropathogen, directly proportional to the rise in the patient’s age. The prevalence of Gram-negative pathogens in the age group of 15-40 years was 138 (43.7%), while that in the age group of 40 years was 180 (56.2%) (Table 1).

**Table 1:** Demographic characteristics of urine culture-positive patients χ2 = 0.72; p = 0.3. χ2 = 53.7; p = 0.001

Variable	Frequency of Gram-negative pathogen		Frequency of Gram-positive pathogen	
Age, years	N	%	N	%
	15–40	138	43.3	29	46.8
˃40	180	56.2	34	54.2
Sex				
Female	213	66.5	39	62.3
Male	105	32.1	24	38.7
Patients				
Out	182	56.1	36	57.5
In	136	42.5	27	43.5

**Figure 1:**
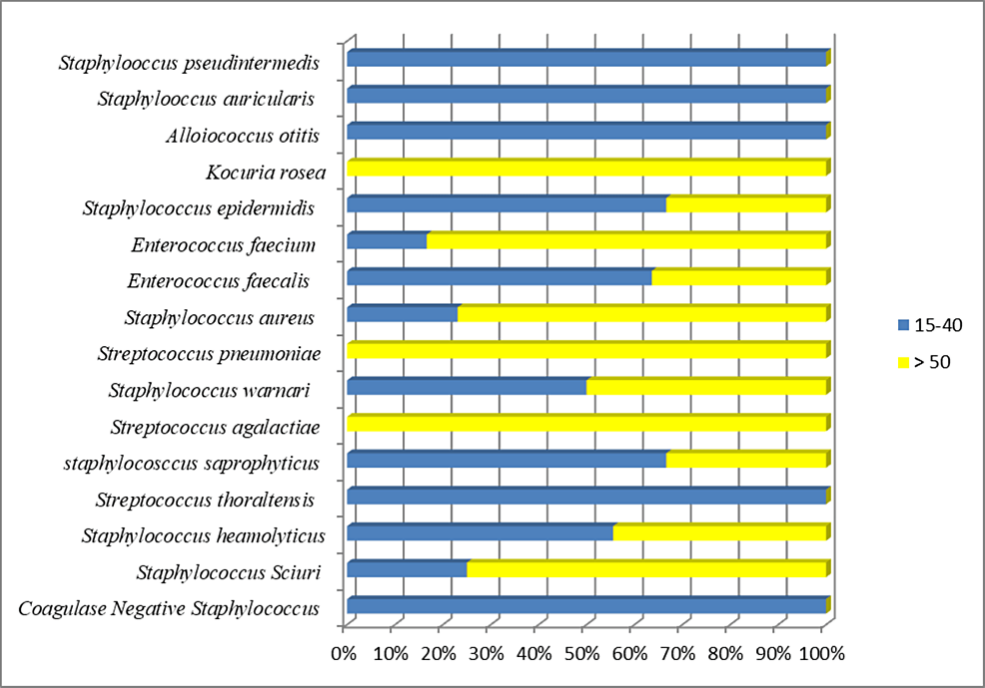
Prevalence of Gram-positive uropathogens by age group age group (15 - 40) and > 40 years old

Gram-positive pathogens accounted for 29 (46.8%) isolates in patients aged 15-40 years and 34 (54.2%) in those aged more than 40 years (Figure 1). Most of the Gram-negative and Gram-positive uropathogens were retrieved from female patients: 213 (66.5%) and 39 (62.3%), respectively, whereas males constituted only 105 (32.1%) and 24 (38.7%) patients (χ2 = 0.72; p = 0.3). The overall prevalence of patients was as follows: outpatients constituted the majority (n = 218, 56.6%), while inpatients numbered 163 (42.7%) (χ2 = 53.7; p = 0.001). A high level of antibiotic resistance was present among inpatients (p<0.001) and in both Gram-negative and Gram-positive isolates (p<0.001).

Profile of uropathogenic bacterial species

**Figure 2:**
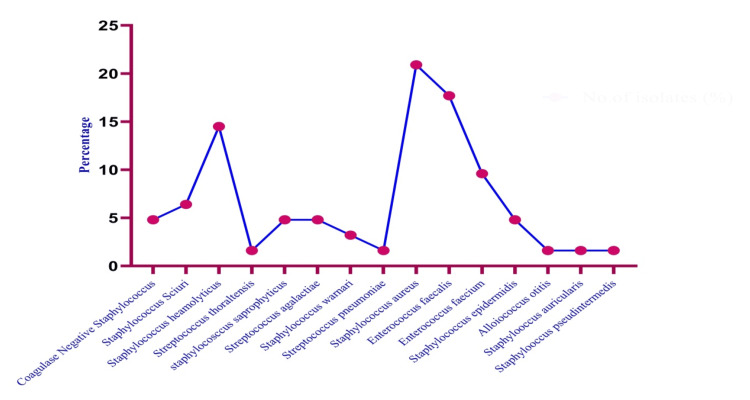
Distributions of Gram-positive uropathogenic bacterial species

**Figure 3:**
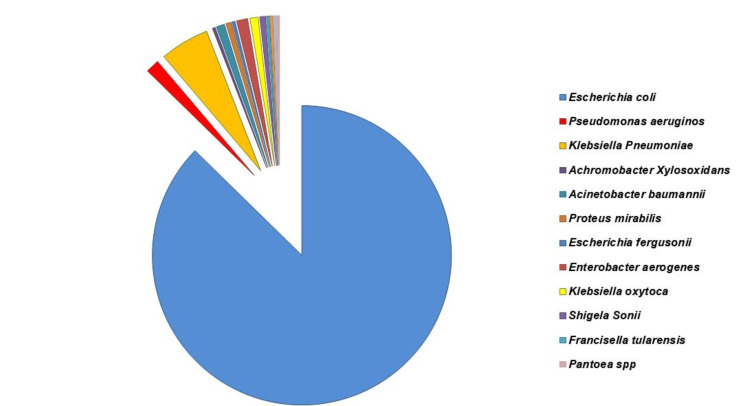
Distributions of Gram-negative uropathogenic bacterial species

Dipstick urinalysis was commonly used in point-of-care diagnosis. Leukocyte esterase (LE) and pyuria (by dipstick analysis) have very high sensitivity and specificity for UTI [>90% as defined by the culture of uropathogens from urine with >100,000 colony forming units (CFU) per ml]. While 63/381 (16.2%) were Gram-positive organisms (Figure 2), the majority of the isolates (318/381, 83.4%) were Gram-negative organisms (Figure 3), and only 13 (3.1%) were mixed, and hence excluded from this study. Twenty-nine different species of uropathogens were isolated. They were classified into two groups (Gram-positive and Gram-negative), based on the frequency for each group, from most to least frequent (Tables 2-3).

**Table 2:** Distribution of Gram-positive uropathogenic bacterial species (n = 63)

Isolated uropathogens (Gram-positive)	N (%)
No growth	3483 (89.8)
Significant growth ((10⁵ CFU/ml)	381 (10.9)
Gram-positive	63 (16.5)
Staphylococcus aureus	13 (20.9)
Enterococcus faecalis	11 (17.7)
Staphylococcus haemolyticus	9 (14.5)
Enterococcus faecium	6 (9.6)
Staphylococcus sciuri	4 (6.4)
Staphylococcus saprophyticus	3 (4.8)
Streptococcus agalactiae	3 (4.8)
Coagulase-negative staphylococci	3(4.8)
Staphylococcus epidermidis	3 (4.8)
Staphylococcus warneri	2 (3.2)
Streptococcus thoraltensis	1 (1.6)
Streptococcus pneumoniae	1 (1.6)
Alloiococcus otitis	1 (1.6)
Staphylooccus auricularis	1 (1.6)
Kocuria rosea	1 (0.3)
Staphylococcus pseudintermedius	1 (1.6)

**Table 3:** Distributions of Gram-negative uropathogenic bacterial species (n = 318)

Isolated uropathogens (Gram-negative)	N (%)
No growth	3483 (89.8)
Significant growth (10⁵ CFU/ml)	381 (10.9)
Gram-negative	318 (83.4)
Escherichia coli	278 (88.8)
Klebsiella pneumoniae	17 (5.4)
Pseudomonas aeruginosa	5 (1.5)
Enterobacter aerogenes	4 (1.2)
Acinetobacter baumannii	3 (0.9)
Klebsiella oxytoca	3 (0.9)
Proteus mirabilis	2 (0.6)
*Shigella* *sonnei*	2 (0.6)
Achromobacter xylosoxidans	1 (0.3)
Escherichia fergusonii	1 (0.3)
Francisella tularensis	1 (0.3)
Pantoea spp	1 (0.3)

Antibiotic susceptibility test

The results of antibiotic susceptibility testing showed different patterns of resistance in the isolated bacteria. Gram-negative isolates were completely resistant to ceftriaxone, cefepime, ceftazidime, nalidixic acid, norfloxacin, gentamicin, ampicillin, and cotrimoxazole. However, they were highly sensitive to polymyxin B, tigecycline, imipenem, and amoxicillin combined with clavulanic acid (Table 4). Among Gram-positive bacteria, most were positive for the cefoxitin screen, except for *S. pneumoniae *(Table 5); some gram-positive bacteria exhibited intermediate negativity, including *Enterococcus faecium (E. faecium) *(3, 50%), coagulase-negative staphylococci (CoNS) 2, 66.7%), and *Enterococcus faecalis (E. faecalis)* (10, 90.9%). Notably, several bacteria displayed high levels of resistance to gentamicin (100%), while others such as CoNS, *Staphylococcus sciuri (S. sciuri), Staphylococcus haemolyticus (S. haemolyticus), Streptococcus thoraltensis (S. thoraltensis), Staphylococcus saprophyticus (S. saprophyticus), Staphylococcus warneri (S. warneri), Streptococcus pneumoniae (S. pneumoniae), and Staphylococcus aureus (S. aureus)* showed no resistance to gentamicin.All the isolates were resistant to benzylpenicillin, ampicillin, and oxacillin.

**Table 4:** Antimicrobial drug resistance in Gram-negative isolated organisms Values presented as n (%) AMP: ampicillin; AMC: amoxicillin-clavulanate; PPT: piperacillin-tazobactam; CRX: ceftriaxone; CEA: cefalexin; CXT: cefotaxime; CTZ: ceftazidime; CTR: cefuroxime; CPM: cefepime; AZT: azithromycin; ENP: ertapenem; IMM: imipenem; MEM: meropenem; AMI: amikacin; GEN: gentamicin; CIP: ciprofloxacin; FUR: Furadantin (nitrofurantoin); TIG: tigecycline; TRS: trimethoprim-sulfamethoxazole E. coli: Escherichia coli; P. aeruginosa: Pseudomonas aeruginosa, K. pneumoniae: Klebsiella Pneumoniae; A. xylosoxidans: Achromobacter xylosoxidans; A. baumannii: Acinetobacter baumannii; P. mirabilis​​​​​: Proteus mirabilis, E. fergusonii​​​​​: Escherichia fergusonii; E. aerogenes​​​​​: Enterobacter aerogenes; K. oxytoca: Klebsiella oxytoca, S. sonnei​​​​​: Shigella sonnei; F. tularensis​​​​​​: Francisella tularensis

Isolates	AMP	AMC	PPT	CRX	CEA	CXT	CTZ	CTR	CPM	AZT	ENP	IMM	MEM	AMI	GEN	CIP	FUR	TIG	TRS
E. coli	278 (100)	1 (100)	232 (83.5)	230 (82.7)	220 (79.1)	267 (96.0)	215 (77.3)	211 (75.9)	156 (56.1)	129 (46.4)	21 (7.6)	13 (4.7)	8 (2.9)	89 (32.0)	83 (29.9)	149 (69.8)	167 (60.1)	169 (60.8)	246 (88.5)
*P*. *aeruginosa*	4 (100)	4 (100)	4 (100)	4 (100)	4 (100)	0.00	0.00	4 (100)	4 (100)	4 (100)	4 (100)	0	0	0	3 (75)	3 (75)	0	3 (75)	3 (75)
K. pneumoniae	15 (100)	15 (100)	5 (29.4)	15 (88.2)	15 (88.2)	10 (58.8)	14 (82.4)	15 (100)	11 (64.7)	2 (11.8)	0	0	0	7 (41.2)	12 (70.6)	6 (35.3)	5 (29.4)	7 (41.2)	14 (82.4)
A. xylosoxidans	1 (100)	1 (100)	1 (100)	1 (100)	1 (100)	0.00	0.00	1 (100)	1 (100)	0	0	0	1 (100)	1 (100)	0	0	0	0	0
A. baumannii	3 (100)	3 (100)	3 (100)	3 (100)	3 (100)	3 (100)	3 (100)	3 (100)	3 (100)	3 (100)	3 (100)	3 (100)	1 (33.3)	2 (66.7)	2 (66.8)	1 (33.3)	1 (33.3)	1 (33.3)	1 (33.3)
P. mirabilis	2 (100)	2 (100)	2 (100)	1 (50)	1 (50)	1 (50)	1 (50)	2 (100)	1 (50)	2 (100)	2 (100)	2 (100)	2 (100)	2 (100)	2 (100)	2 (100)	2 (100)	2 (100)	1 (50)
E. fergusonii	1 (100)	1 (100)	0.00	1 (100)	1 (100)	0.00	1 (100)	1 (100)	1 (100)	1 (100)	1 (100)	0	0	0	1 (100)	1 (100)	0	0	0
E. aerogenes	4 (100)	4 (100)	3 (75)	4 (100)	4 (100)	4 (100)	4 (100)	4 (100)	4 (100)	4 (100)	4 (100)	0	4 (100)	3 (75)	3 (75)	0	0	0	0
K. oxytoca	3 (100)	3 (100)	0.00	2 (66.7)	2 (66.7)	3 (100)	3 (100)	3 (100)	3 (100)	0	0	0	1 (33.3)	2 (66.7)	3 (100)	2 (66.7)	1 (33.3)	0	0
S. sonnei	2 (100)	2 (100)	0.00	2 (100)	2 (100)	2 (100)	2 (100)	2 (100)	0	0	2 (100)	0	0	0	0	0	0	0	2 (100)
F. tularensis	1 (100)	1 (100)	1 (100)	1 (100)	1 (100)	0.00	0.00	1 (100)	1 (100)	1 (100)	1 (100)	0	0	0	1 (100)	1 (100)	1 (100)	1 (100)	1 (100)
Pantoea spp	1 (100)	1 (100)	1 (100)	1 (100)	1 (100)	1 (100)	0.00	1 (100)	1 (100)	1 (100)	1 (100)	0	0	0	1 (100)	1 (100)	0	1 (100)	1 (100)

**Table 5:** Antimicrobial drug resistance in gram-positive isolated organisms Values presented as n (%) CoNS: coagulase-negative staphylococci; S. sciuri: Staphylococcus sciuri; S. haemolyticus​​​​​: Staphylococcus haemolyticus; S. thoraltensis​​​​​​: Streptococcus thoraltensis; S. saprophyticus​​​​​​: staphylococcus saprophyticus; S. agalactiae​​​​​​: Streptococcus agalactiae; S. warneri​​​​​​: Staphylococcus warneri; S. pneumoniae​​​​​​: Streptococcus pneumoniae; S. aureus​​​​​​: Staphylococcus aureus; E. faecalis​​​​​​: Enterococcus faecalis; E. faecium​​​​​​: Enterococcus faecium; S. epidermidis​​​​​​: Staphylococcus epidermidis; A. otitidis: Alloiococcus otitidis; K. rosea: Kocuria rosea; S. auricularis: Staphylococcus auricularis; S. pseudintermedius: Staphylococcus pseudintermedius

Isolates	Cefoxitin screen		Gentamicin high-level resistance		Inducible clindamycin resistance		Benzylpenicillin		Ampicillin		Oxacillin		Levofloxacin		Erythromycin		Nitrofurantoin		Linezolid
CoNS	2 (66.7)		0		0		3 (100)		3 (100)		3 (100)		2 (66.7)		2 (66.7)		1 (33.3)		0
S. sciuri	4 (100)		0		0		4 (100)		4 (100)		4 (100)		4 (100)		4 (100)		2 (50)		0
S. haemolyticus	9 (100)		0		4 (44.4)		9 (100)		9 (100)		9 (100)		9 (100)		7 (77.8)		0		0
S. thoraltensis	1 (100)		0		1 (100)		1 (100)		1 (100)		1 (100)		1 (100)		1 (100)		0		0
S. saprophyticus	3 (100)		0		2 (66.6)		3 (100)		3 (100)		3 (100)		3 (100)		3 (100)		0		0
S. agalactiae	3 (100)		1 (33.3)		0		3 (100)		3 (100)		3 (100)		3 (100)		3 (100)		0		0
S. warneri	2 (100)		0		0		2 (100)		2 (100)		2 (100)		3 (100)		0		0		0
S. pneumoniae	0		0		0		1 (100)		1 (100)		1 (100)		1 (100)		0		0		0
S. aureus	13 (100)		0		4 (30.8)		13 (100)		13 (100)		13 (100)		13 (100)		13 (100)		5 (38.5)		2 (15.4)
E. faecalis	10 (90.9)		3 (27.3)		1 (9.1)		11 (100)		11 (100)		11 (100)		8 (72.7)		11 (100)		6 (54.5)		8 (72.7)
E. faecium	3 (50)		5 (83.3)		1 (16.7)		6 (100)		6 (100)		6 (100)		1 (16.7)		2 (33.3)		1 (16.7)		6 (100)
S. epidermidis	3 (100)		3 (100)		0		3 (100)		3 (100)		3 (100)		1 (33.3)		1 (33.3)		1 (33.3)		0
A. otitidis	1 (100)		1 (100)		0		1 (100)		1 (100)		1 (100)		0		1 (100)		0		1 (100)
K. rosea	1 (100)		1 (100)		1 (100)		1 (100)		1 (100)		0.00		0.00		1 (100)		1 (100)		0
S. auricularis	1 (100)		1 (100)		0		1 (100)		1 (100)		1 (100)		0		1 (100)		0		1 (100)
S. pseudintermedius	1 (100)		1 (100)		0		1 (100)		1 (100)		1 (100)		0		1 (100)		0		1 (100)
Isolates		Daptomycin		Teicoplanin		Vancomycin		Tetracycline		Tigecycline		Nitrofurantoin		Fusidic acid		Mupirocin		Trimethoprim/sulfamethoxazole	
CoNS		1 (33.3)		1 (33.3)		1 (33.3)		2 (66.7)		1 (33.3)		1 (33.3)		2 (66.7)		66.7/2		66.7/2	
S. sciuri		3 (75)		3 (75)		3 (75)		3 (75)		2 (50)		2 (50)		4 (100)		50/2		50/2	
S. haemolyticus		0		2 (22.2)		1 (11.1)		5 (55.6)		0		0		88.8/8		0		44.4/4	
S. thoraltensis		0		0		0		1 (100)		0		0		1 (100)		0		0	
S. saprophyticus		0		0		0		3 (100)		0		0		3 (100)		0		0	
S. agalactiae		0		0		0		3 (100)		3 (100)		0		3 (100)		0		0	
S. warneri		0		2 (100)		0		2 (100)		2 (100)		0		2 (100)		0		0	
S. pneumoniae		0		1 (100)		0		1 (100)		1 (100)		0		1 (100)		0		0	
S. aureus		1 (7.7)		11 (84.6)		4 (30.8)		9 (69.2)		1 (100)		5 (38.5)		13 (100)		38.5/5		84.6/11	
E. faecalis		7 (63.6)		9 (81.8)		10 (90.9)		8 (72.7)		7 (63.6)		6 (54.5)		0		0		45.5/5	
E. faecium		2 (33.3)		6 (100)		6 (100)		4 (66.7)		1 (16.7)		1 (16.7)		6 (100)		0		100/6	
S. epidermidis		1 (33.3)		0		0		1 (33.3)		0		1 (33.3)		2 (66.7)		0		33.3/1	
*A. *otitidis		0		1 (100)		0		0		1 (100)		0		0		0		0	
S. auricularis		0		1 (100)		0		0		1 (100)		0		0		0		0	
S. pseudintermedius		0		1 (100)		0		0		1 (100)		0		0		0		0	
K. rosea		0		1 (100)		0		1 (100)		1 (100)		0.00		0.00		1 (100)		1 (100)	

In addition, different levels of resistance to levofloxacin were found among bacteria, except the newly reported Gram-negative bacteria - *Francisella tularensis (F. tularensis​​​​​​), Pantoea spp*., and* Escherichia fergusonii (E. fergusonii​​​​​) -* which were found to be sensitive to levofloxacin. High levels of resistance to erythromycin were found in all bacteria except for* S. warneri and S. pneumoniae*, which are completely sensitive to erythromycin. *Alloiococcus otitidis​​​​​​​ (A. otitidis​​​​​​​), Staphylococcus auricularis *(S. auricularis​​​​​​​)*, and Staphylococcus pseudintermedius (S. pseudintermedius)*, were resistant to linezolid (1, 100%), tigecycline (1, 100%), and teicoplanin (1, 100%). *E. faecium* had the highest rate of antibiotic resistance to vancomycin (6, 100%), followed by CoNS, *S. Sciuri, S. haemolyticus, S. aureus, and E. faecalis:* ​​​​​​​(1, 33.3%), (3, 75%), (1, 11.1%), (4, 30.8%), and (10, 90.9%) respectively. *S. thoraltensis, S. saprophyticus, S. agalactiae, S. warneri, and S. pneumonia,* showed the highest antibiotic resistance to tetracycline (100%), while* A. otitidis, S. auricularis, and S. pseudintermedius* showed an increase in antibiotic sensitivity (100%). However, *S. haemolyticus, S. thoraltensis, S. saprophyticus, and S. epidermidis* showed no antibiotic resistance to tigecycline, in contrast to the rest of the isolates, which were observed to have varying levels of antibiotic resistance (Table 4).

*E. faecalis *displayed preserved rates of resistance to nitrofurantoin (6, 54.5%), while a decrease in the resistance was observed in *S. thoraltensis, S. saprophyticus, S. agalactiae, S. warneri, and S. pneumonia*e (0%). *S. thoraltensis, S saprophyticus, S. agalactiae, S. warneri, and S. pneumoniae* showed the highest resistance to fusidic acid (100%) overall, while newly reported isolates and *E. faecalis* displayed no signs of antibiotic resistance (0%). Different levels of antibiotic resistance to mupirocin were observed in CoNS, *S. sciuri, and S. aureus:* (2, 66.7%), (2, 50%), and (5, 38.5%) respectively; however, the other isolates showed 0% antibiotic resistance. *E. faecium* showed the highest resistance rates to trimethoprim/sulfamethazole (6, 100%), in contrast to newly reported bacteria that showed no resistance.

*E. coli* was the most frequently encountered uropathogenic (278, 88.8%), and it showed the overall highest rate of resistance among the studied antibiotics to ampicillin, followed by amoxicillin-clavulanic acid (278, 100%), and trimethoprim/sulfamethoxazole (246, 88.5%). Maintained sensitivity was observed to carbapenems-meropenem (8, 2.9%), imipenem (13, 4.7%), ertapenem (21, 7.6%), and gentamicin (83, 29.9%) (Table 5). *Klebsiella spp*. was the second-most frequent pathogen studied (17, 5.4%), and it showed the overall highest resistance to ampicillin, amoxicillin/clavulanic acid (100%), ceftriaxone (100%), cefuroxime and cefuroxime axetil (15, 88.2%).

No resistance was observed in the case of carbapenems (0%). *Pseudomonas* species accounted for the majority of antibiotic resistance investigated. Resistance rates of 100% were found for the following medications: ertapenem, ampicillin, amoxicillin/clavulanic acid, piperacillin/tazobactam, cefuroxime, axetil ceftriaxone, cefepime, and aztreonam, while there was 0% resistance to nitrofurantoin; imipenem, meropenem, amikacin, cefoxitin, and ceftazidime are among the drugs with reduced resistance rates. Based on the results, most *Enterobacter aerogenes (E. aerogenes)* isolates (98.8%) exhibited high levels of resistance to all tested antibiotics. However, four (1.2%) showed no resistance to imipenem, ciprofloxacin, nitrofurantoin, tigecycline, trimethoprim, or sulfamethoxazole. Both *Proteus mirabilis *(P. mirabilis​​​​​) ​​​​​​​(2, 0.3%) and *Acinetobacter baumannii* *(A. baumannii)* (3, 0.9%) showed preserved rates of resistance to all tested antibiotics and are multidrug resistant. The strain of *Klebsiella oxytoca (K. oxytoca)* ​​​​​​​(3, 0.9%) exhibited the highest ampicillin (3, 100%) and gentamicin (3, 100%) resistance rates.

**Figure 4:**
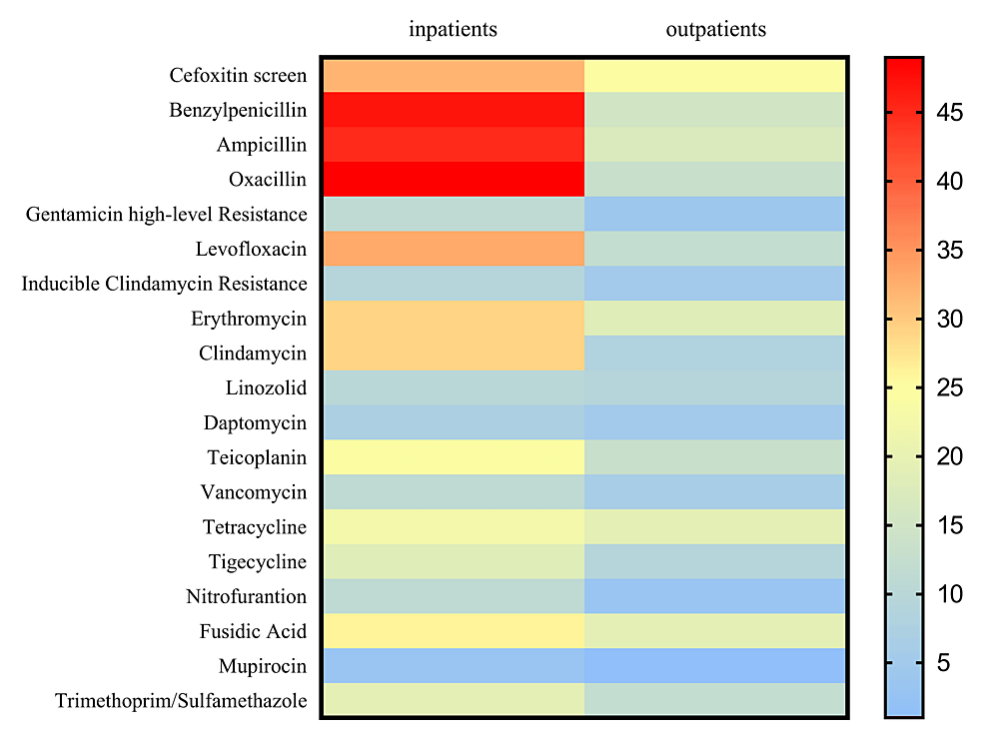
Mapping antibiotic resistance (Gram-positive): a heatmap analysis of prevalence in community and hospitalized patients t = 20.28; df = 18; p<0.0001

**Figure 5:**
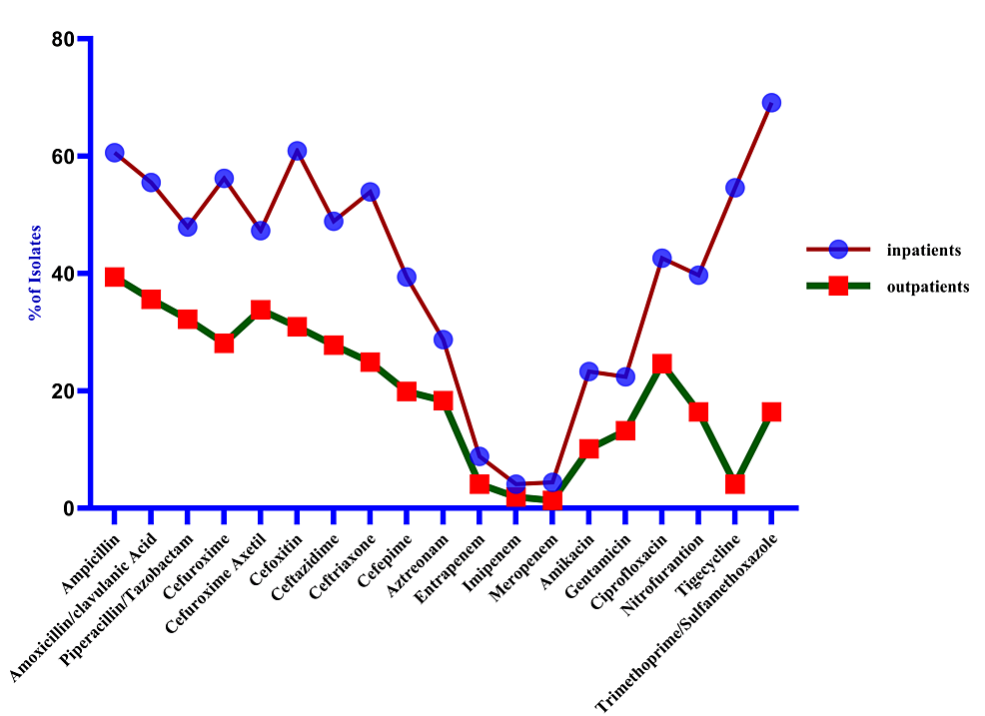
Antibiotic resistance (Gram-negative): an analysis of prevalence in community and hospitalized patients P = 0.0001

K. oxytoca showed reduced resistance to trimethoprim/sulfamethoxazole, ertapenem, imipenem, aztreonam, and piperacillin/tazobactam. In Gram-positive pathogens (Figure 4), the present study demonstrated more antibiotic resistance among inpatients to benzylpenicillin (47, 100) ampicillin (45, 100), and oxacillin (49, 100), whereas, in community patients, a high ratio of resistance was found regarding linezolid (9, 47.4). According to results in gram-negative isolates, increased resistance among hospitalized patients was present in trimethoprim/sulfamethoxazole (69.1%), ampicillin (60.6%), cefoxitin (193, 60.9), and amoxicillin/clavulanic acid (55%). In contrast, lower rates were found regarding imipenem (4.1%); in community patients, there was a high frequency of resistance to ampicillin (39.6%) and amoxicillin/clavulanic acid (35.4%) while there was a high frequency of resistance to imipenem (1.4%) and meropenem (1.3%) (Figure 5).

The least frequent uropathogens (newly reported) - *E. fergusonii* (1, 0.3%), *F. tularensis* (1, 0.3%), and *Pantoea spp*. (0.3%) - showed the highest resistance rate to all antibiotics tested, except for imipenem, meropenem, and amikacin. *Kocuria rosea *(K. rosea) (0.3%), a newly reported uropathogenic bacteria, showed an increase in its susceptibility to the majority of tested antibiotics in general. While the least alarming scenarios were observed in the case of cefoxitin, ceftazidime, and amikacin, *Shigella sonnei (S. sonnei)* ​​​​​(2, 0.6%) revealed conserved rates of resistance to most of the tested antibiotics, excluding piperacillin/tazobactam, cefepime, aztreonam, imipenem, meropenem, amikacin, and gentamicin, ciprofloxacin, nitrofurantoin, and tigecycline. *Achromobacter xylosoxidans (A. xylosoxidans)* (1, 0.3%) preserved resistance to cefepime, meropenem, and amikacin while showing sensitivity to aztreonam, ertapenem, imipenem, amikacin, gentamicin, ciprofloxacin, nitrofurantoin, and tigecycline.

## Discussion

The gold standard for UTI diagnosis is the bacterial culture of the urine. UTI is one of the most prevalent diseases, with diverse etiological agents. It is common in most regions of Iraq and remains a major health problem in many developing countries [13]. An analysis of our current findings showed that 318 (9.8%) of the samples were positive for microorganism growth in UTI. Results that contrast with ours were found in Duhok Province, Iraq (80%) [14], and in Babylon City, Iraq (89.72%) [15]. Females (61.3%) were more infected than males in our samples, which aligns with studies by other researchers [1,14]. However, in other studies, no significant difference was observed between bacterial infections of the female and male urinary systems [16]. Their high predisposition to contract infection is due to their genital anatomy. Regarding age groups, the highest prevalence was found in the elderly group (55.7%), which could be attributed to several factors such as urinary tract abnormalities, disability, decreased immune response, prostate disorders in men, and hormonal changes in women. Among the detected UTI strains, 15.7% were Gram-positive and 81.2% were Gram-negative [14]. Uropathogenic Gram-negative bacterial infection is one of the most prevalent diseases with diverse etiological agents in most regions of Iraq and remains a major health problem in many developing countries [17].

The impact of the COVID-19 pandemic has led to changes in the field of epidemiology and the spread of numerous contagious infections, both in hospital and outpatient settings. AMR is a silent epidemic that gathered strength through the pandemic [18]. Many studies have analyzed the effect of the pandemic on AMR in the setting of UTI [18]. The reason behind this is multifactorial and is chiefly interconnected with the large amounts of antimicrobial agent consumption for prophylactic management or treatment of co- or secondary infections in COVID-19 patients [19]. With improved awareness about antimicrobial stewardship, most hospitals are reassessing the logical use of antibiotics and updating their antibiogram intermittently. However, in community settings, there is still a long way to go [19].

In the context of the impact of COVID-19 waves on all levels of healthcare, it is critical to reassess and modify research to determine the true effects of this global epidemic on UTIs and patterns of AMR. The most common uropathogenic bacteria found in urine samples are Gram-negative. There was also strong evidence against Gram-positive UTI infection [20]. This paper presents data from patients of both sexes aged 15-75 years. In 10.08% (382/3877) of the urine culture samples, microbiological evidence of infection was found, which is similar to another study [2]. In contrast, some studies have reported higher prevalence rates of 45% and 70.83% [21]. The lower relative prevalence in this study could be because patients were treated with antibiotics before arriving at the institute for diagnosis, and the pathogens were killed or inhibited. Variations in research population characteristics, environmental factors, and techniques may help explain this discrepancy [22]. In this study, females (65.9%) significantly outnumbered males in terms of positivity. Regarding age, the highest prevalence was found in the elderly group (55.7%).

In concordance with previous research [23], our findings indicate that 15.7% were Gram-positive while 81.2% were Gram-negative pathogens [9]. *E. coli* accounted for the majority of infections (88.8%). In fact, almost all studies have found that this bacterium is the most common uropathogenic across all continents, in varying degrees. This result is in agreement with other studies [24]. Similarly, high rates of *E.coli* incidence were detected in Western European countries such as France and Austria, reporting rates over 65% in the studied populations [25]. The second most common Gram-negative bacteria was *Klebsiella spp.*, representing (17%) of the total strains; similar findings have been reported elsewhere [25]. This is comparable with other findings in other regions in the Middle East, such as Turkey (11.2%) [26]. *Pseudomonas aeruginos* accounted for 1.5%), similar to another study [25], while *Enterobacter aerogenes *represented 1.2%, which is lower than what was reported by Antimicrobial Resistance Collaborators [22], holding third and fourth places, respectively. In terms of the frequency of uropathogens with interchangeable proportions, the study suggested that the emergence of carbapenem-resistant Enterobacteriaceae colonization increased from 6.7% to 50% in the wake of COVID-19 [27].* S. aureus* and* E. faecalis* showed the highest rates among Gram-positive isolates, aligning with a few other studies [20,22].

Pathogens that are isolated from UTI patients show exceedingly great levels of AMR to frequently used treatments (Table 5). Among Gram-negative bacteria, the highest percent of resistance towards first-line antibiotics was found toward amoxicillin and amoxicillin/clavulanic acid; these results are in agreement with those of other studies [28]. However, amoxicillin and co-trimoxazole resistance rates observed were higher in comparison to other reports. Hence, empirical therapy with these antibiotics seems inadequate and should be avoided. Our results show a substantial increase in the resistance to quinolones among common isolates, an observation also reported by others [28]. The quinolones were the least effective agents against all the uropathogens encountered during the study period. This observation positively correlates with the previous reports since the mechanism of action of these quinolones is almost the same; the emergence of resistance against one will also affect the activity of other quinolones [29]. This contrasts with other studies, which have reported higher susceptibility to fluoroquinolone [29].

Carbapenem antibiotics such as meropenem and imipenem were the most effective drugs against *E. coli *and *K. pneumoniae*. Amikacin was found to be effective against *P. aeruginos*. Similar results were demonstrated by Mareș et al. [25]. Carbapenem was also found to be active against* P. aeruginos*; while this contrasts with other studies, our results otherwise found this bacterium presented the most dramatic falls in the sensitivity to ciprofloxacin, which aligns with Mareș et al. [25]. A lower resistance was observed for nitrofurantoin in our study, which validates the findings of the study by Mareș et al. [25]. The most common urease-producing bacteria, *Proteus spp.*, showed higher resistance and significant growth against all antibiotics, which agrees with the results of Altamimi et al. [30]. The same problem of MDR was demonstrated by *A. baumannii*, aligning with the findings of Meawed et al. [31]. *E. coli, P. aeruginos. S. pneumoniae, A. baumannii, and P. mirabilis *were found to be resistant to gentamicin and ciprofloxacin. This result is dissimilar to the result documented by Kashyap et al. [32]. The antibiogram resistance profile of Gram-positive bacteria is summarized in Table 4, and The prevalence of MDR in various isolates is illustrated in Tables 4-5.

*Enterococcus spp*. showed high resistance (100%) to clindamycin and teicoplanin, which is similar to the findings of Abdel Gawad et al. [33]. Gram-positive bacteria showed considerably high resistance rates to Antibiotics in this study, which is incompatible with the investigation conducted by Ahmed et al. [23]. Most *S. aureus* and *Enterococcus spp *were found resistant to β-lactam antibiotics. These results are in agreement with other studies [28]. Ampicillin resistance was recorded to be 83.3-100% and 76.9-100% in hospitalized and community-related samples, respectively, while meropenem susceptibility was limited in both groups. Ampicillin resistance was shown to be prevalent in samples obtained from the community and hospitalized samples, but meropenem resistance was limited in both study groups, as found by Adugna et al. [3].

Limitations

This study has a few limitations. Primarily, our study's scope was limited due to the small quantity of urine culture. As the study samples were collected from a single center, the generalizability of the results is limited. Additionally, due to inadequate laboratory facilities, we could not examine anaerobic bacteria. Moreover, misdetection of antibiotic resistance-encoding genes, due to the lack of molecular lab was another shortcoming. Another limitation pertains to urine contamination from vaginal or periurethral microorganisms, which was not considerably decreased by the collection method (midstream "clean catch"). Furthermore, treatment outcomes could not be recorded in inpatient medical records because of access restrictions imposed during the COVID-19 pandemic. Some cases may have been missed out; for example, patients undergoing therapy without having a urine culture taken or getting antibiotics beforehand. Also, only a small number of non-*E. coli* infections were included because of their lower frequency rates. Moreover, because the study was done during the start of the COVID-19 pandemic, which only lasted two years, it becomes difficult to pinpoint any notable variations in vulnerability profiles during that specific time frame.

Strengths

A key strength of our study lies in the fact that it is the first investigation examining antimicrobial agent susceptibility patterns associated with COVID-19 conducted in our community. Moreover, since we included all urine culture samples that were collected and submitted to the hospital laboratory throughout the pandemic, we were able to extrapolate the findings to a similar setting.

Rare, newly emerged, and underreported polymicrobial UTI sources

We included several particular instances of rare, emergent, or underreported Gram-positive bacteria, including species of *Alloiococcus otitis, Staphylococcus auricularis, Staphylococcus pseudintermedius, Kocuria rosea, and Staphylococcus warneri*. In addition to Gram-negative bacteria, we included species of *Francisella tularensis, Pantoea spp, and Escherichia fergusonii,* because these organisms may often go unnoticed as causes of UTI: "The potential for misinterpretation arises when distinguishing phenotypic characteristics are lacking. Additionally, there is a risk of overlooking significant growth by dismissing it as mere 'microbiota contamination'.'' Also, there are no studies that include all types of bacteria causing UTIs, and most studies are limited to specific bacteria. Generally, this tends to increase bacterial resistance to most of the tested antibiotics.

## Conclusions

This study explored how the COVID-19 pandemic affected the residual antibiotic profiles and the pathogenic organisms that patients were exposed to.* E. coli *and* Klebsiella spp*. were the microorganisms most frequently linked to the epidemiology of urogenital illnesses during the pandemic. *Staphylococcus spp.* was the most prevalent Gram-positive bacteria. In the setting of COVID-19, several new varieties of bacterial organisms causing infections of the urinary tract have emerged, and data collected from the hospital indicate that the rate of antibiotic resistance was lower in earlier years. In addition, it is important to note that empirical antibiotic therapy should be based on regional patterns of antibiotic sensitivity rather than global recommendations for COVID-19 patients, which involve administering broad-spectrum antibiotics. Additionally, individuals experiencing MDR should be advised to proceed with caution when selecting antibiotics for UTI, as the overuse of broad-spectrum drugs for such infections can lead to antibiotic resistance. Hospital-acquired UTIs are more prevalent than community-acquired infections due to growing resistance to antibiotics, including ampicillin, posing a serious public health risk due to increased sensitivity to drugs such as meropenem and imipenem.
